# Novel 3D heart left ventricle muscle segmentation method for PET-gated protocol and its verification

**DOI:** 10.1007/s12149-019-01373-6

**Published:** 2019-06-01

**Authors:** Tomasz Kubik, Krzysztof Kałużyński, Cyrill Burger, Alessandro Passeri, Selene Margiacchi, Paola Saletti, Rita Bonini, Elena Lorenzini, Roberto Sciagrà

**Affiliations:** 1 Faculty of Mechatronics, Institute for Metrology and Biomedical Engineering, Warsaw University of Technology, ul. św. Andrzeja Boboli 8, 02-525 Warsaw, Poland; 2 Pmod Technologies LLC, Sumatrastrasse 25, 8006 Zurich, Switzerland; 3 Nuclear Medicine, ECBSD, University of Florence, Largo Brambilla 3, 50134 Florence, Italy; 4 Medical Physics, AOU Careggi, Largo Brambilla 3, 50134 Florence, Italy; 5 Nuclear Medicine, ASL Nord Ovest, Via Risorgimento 18, 54100 Massa, Italy; 6 Medical Physics, ASL Nord Ovest, Piazza Sacco e Vanzetti 5, 54033 Carrara, Italy

**Keywords:** Segmentation method, Gated PET imaging, Volume quantification, left ventricle, cardiology

## Abstract

**Objective:**

The aim of this study was to propose and verify a universal method of left ventricular myocardium segmentation, able to operate on heart gated PET data with different sizes, shapes and uptake distributions. The proposed method can be classified as active model method and is based on the BEAS (B-spline Explicit Active Surface) algorithm published by Barbosa et al. The method was implemented within the Pmod PCARD software package. Method verification by comparison with reference software and phantom data is also presented in the paper.

**Methods:**

The proposed method extends the BEAS model by defining mechanical features of the model: tensile strength and bending resistance. Formulas describing model internal energy increase during its stretching and bending are proposed. The segmentation model was applied to the data of 60 patients, who had undergone cardiac gated PET scanning. QGS by Cedars-Sinai and ECTb by Emory University Medical Centre served as reference software for comparing ventricular volumes. The method was also verified using data of left ventricular phantoms of known volume.

**Results:**

The results of the proposed method are well correlated with the results of QGS (slope: 0.841, intercept: 0.944 ml, *R*^2^: 0.867) and ECTb (slope: 0.830, intercept: 2.109 ml, *R*^2^: 0.845). The volumes calculated by the proposed method were very close to the true cavity volumes of two different phantoms.

**Conclusions:**

The analysis of gated PET data by the proposed method results in volume measurements comparable to established methods. Phantom experiments demonstrate that the volume values correspond to the physical ones.

## Introduction

Segmentation is one of the most important steps of image processing in cardiac diagnostics. It allows volume calculation [1, 2] and plays an important role when deriving regional time activity curves for signal modeling [3–6] or for heart muscle strain approximation [7, 8]. The aim of this study was to propose a flexible method for human gated PET left ventricular myocardium segmentation, which operates on heart data of different sizes, shapes and uptake distributions.

The proposed method can be classified as an active model segmentation algorithm. The idea of active model segmentation is to modify position and shape of the model, so that it best satisfies some energy functions [9]. The classic formulation of active model segmentation was described by Kass et al. [10]. They proposed a model restricted by its stiffness and elasticity, which is deformed in the energy field derived from image gradient. The idea represents a group of methods called edge-based active models. They were further developed in several works, e.g. [11–14]. Region-based active models represent another approach, which is less sensitive to image noise. It was first proposed by Chan and Vese [15], who defined the model as dependent on the energy derived from differences in pixel mean values inside and outside the model boundary. Based on their work, many features and energy functions were formulated [16–18]. To increase the method efficiency, Lankton and Tannenbaum [19] proposed a localized approach, in which just the neighborhood of the model is taken into account during image feature analysis. Such a solution connected with an explicit form of model description [20, 21] was used by Barbosa et al., developing the B-spline Explicit Active Segmentation (BEAS) algorithm [22, 23]. They described in a spherical coordinate system a B-spline surface model stretched on a set of nodes, whose angle coordinates are locked and their positions described by only one explicit coordinate—the radius (*r*) [24]. To make the method more effective in epicardium and endocardium segmentation, an extended definition of the BEAS model energy is proposed in this study.

The model was implemented and tested using the software package Pmod PCARDP, Pmod Technologies LLC [25]. This paper presents the model definition, describes its application to human gated PET data as well as physical phantom data and presents the results of left ventricular volume measurements compared to the ones obtained using the reference software packages: QGS—quantitative gated SPECT [26] and Emory cardiac toolbox ECTb [27].

## Materials and methods

### Model energy definition

Barbosa et al., proposed in [22] the following model description:

1 $$r = \psi \left( {\varphi ,\theta } \right) = \mathop \sum \limits_{{k \in {\mathbb{R}}^{2} }} c[k]\beta^{d} \left( {\frac{p}{h} - k} \right),$$

where $\varphi$ and $\theta$ are angle coordinates and *r* is a radius coordinate in the spherical coordinate system with origin inside the model, $\beta^{d}$ uniform symmetric B-spline function in two-dimensional space, $c[k]$ the B-spline coefficients, and $p$ is a point of coordinates $\left( {\varphi ,\theta } \right)$. Model nodes *k* are located on a rectangular grid with spacing *h*.

The energy function, which drives the model shape modification, was proposed as follows:

2 $$E_{\text{I}} = \mathop \int \limits_{\varGamma }^{{}} F\left( {x,H_{\phi } (x)} \right){\text{d}}x = \mathop \int \limits_{\varGamma }^{{}} f_{\text{in}} \left( {I\left( x \right)} \right)H_{\phi } (x) + f_{\text{out}} \left( {I\left( x \right)} \right)\left( {1 - H_{\phi } (x)} \right){\text{d}}x,$$

where $H_{\phi } (x)$ is the Heaviside function [28], $\varOmega$ an image space and $f_{\text{in}}$ and $f_{\text{out}}$ are formulae defining energy inside and outside the model:

3 $$\left\{ {\begin{array}{*{20}c} {f_{\text{in}} \left( {I\left( x \right)} \right) = \left( {I\left( x \right) - u} \right)^{2} } \\ {} \\ {f_{\text{out}} \left( {I\left( x \right)} \right) = \left( {I\left( x \right) - v} \right)^{2} } \\ \end{array} } \right.,$$

where $I\left( x \right)$ is the pixel intensity in point $x$, and $u$ and $v$ are mean pixel intensities inside and outside the model. The authors proved that the direction of the function (2) minimization can be determined from its gradient with respect to B-spline coefficients [22]:

4 $$\frac{{\partial E_{\text{I}} }}{\partial c\left[ k \right]} = \mathop \int \limits_{\varGamma }^{{}} \overline{g} \left( x \right)\beta^{d} \left( {\frac{x}{h} - k} \right){\text{d}}x,$$

where $\varGamma$ is the model surface and

5 $$\bar{g}\left( x \right) = f_{\text{in}} \left( {I\left( x \right)} \right) - f_{\text{out}} \left( {I\left( x \right)} \right).$$

Finally, when running the segmentation algorithm, model shape is modified iteratively by B-spline coefficient recalculation for minimization of *E*_I_:

6 $$c\left[ k \right]^{(t + 1)} = c\left[ k \right]^{(t)} + \lambda \frac{{\partial E_{I} }}{{\partial c\left[ k \right]^{(t)} }},$$

where $t$ is an iteration number and $\lambda$ is a step scaling factor defined by the user as one of the method parameters. The above formulas express the global idea of the BEAS algorithm. Authors have also presented a localized version of the formulas, which take into account only pixel values in the neighborhood of the model nodes. This makes an algorithm more precise and faster.

The model description is extended in this work by the features analogous to mechanical tensile strength and bending resistance. These additional elements are called internal model energy in what follows.

According to the model definition (1), surface nodes can move only along lines defined by their angle coordinates, so model stretching corresponds to increasing the difference in $r$ values for the neighboring nodes. We propose the following formulation of the energy *E*_s_ resulting from model stretching:

7 $$E_{\text{S}} = \mathop \int \limits_{\varGamma }^{{}} \alpha \left( x \right)\left| {\frac{{{\text{d}}\varGamma }}{{{\text{d}}x}}} \right|^{2} {\text{d}}x = \mathop \int \limits_{\varGamma }^{{}} \alpha \left( {p^{*} } \right)\left| {\psi \left( x \right) - \psi \left( {x - h} \right)} \right|^{2} {\text{d}}x,$$

where $\alpha \left( x \right)$ is a constant corresponding to model stiffness in the point $x$. Energy for a node defined by the angle coordinates can be expressed as the superposition of the finite differences of $\psi \left( x \right)$ in both directions $\left( {\left( {r\left( {\varphi ,\theta } \right) - r\left( {\varphi - h,\theta } \right)} \right)/h} \right)^{2}$ and $\left( {\left( {r\left( {\varphi ,\theta } \right) - r\left( {\varphi ,\theta - h} \right)} \right)/h} \right)^{2}$ [29]. Minimization direction and node speed are then determined from the energy gradient:

8 $$\overline{{E_{\text{S}} }} = \frac{{{\text{d}}E_{\text{s}} }}{{{\text{d}}x}} = \frac{{\partial E_{\text{s}} }}{\partial \varphi } + \frac{{\partial E_{\text{s}} }}{\partial \theta } = \frac{{\partial^{2} \varGamma }}{{\partial \varphi^{2} }} + \frac{{\partial^{2} \varGamma }}{{\partial \theta^{2} }}.$$

Stretching energy defined in this way will be minimal, when the model takes the shape of a sphere.

The definition of the bending energy *E*_B_ is expressed using a second derivative of the model function:

9 $$E_{\text{B}} = \mathop \int \limits_{\varGamma }^{{}} \beta \left( x \right)\left| {\frac{{{\text{d}}^{2} \varGamma }}{{{\text{d}}x^{2} }}} \right|^{2} {\text{d}}x\; = \;\mathop \int \limits_{\varGamma }^{{}} \beta \left( x \right)\left| {\psi \left( {x + h} \right) - 2\psi \left( x \right) + \psi \left( {x - h} \right)} \right|^{2} {\text{d}}x,$$

where $\beta \left( x \right)$ is a constant corresponding to model bending resistance in point $x$. Energy for the node defined by the angle coordinates can be expressed as the superposition of the second-order finite differences of $\psi \left( x \right)$.

Analogous to (8), direction and speed of model adjustments leading to minimal energy $E_{\text{E}}$ are determined from fourth derivative of the model with respect to its parameters (9):

10 $$\overline{{E_{\text{B}} }} = \frac{{{\text{d}}E_{\text{B}} }}{{{\text{d}}x}} = \frac{{\partial E_{\text{B}} }}{\partial \varphi } + \frac{{\partial E_{\text{B}} }}{\partial \theta } = \frac{{\partial^{4} \varGamma }}{{\partial \varphi^{4} }} + \frac{{\partial^{4} \varGamma }}{{\partial \varphi^{2} \partial \theta^{2} }} + \frac{{\partial^{4} \varGamma }}{{\partial \theta^{4} }}.$$

Overall energy of the proposed model contains the following components: image energy defined in BEAS method $E_{\text{I}}$ (2), stretching energy $E_{\text{S}}$ (7) and bending energy $E_{\text{B}}$ (9):

11 $$E = E_{\text{I}} + E_{\text{s}} + E_{\text{B}} .$$

Direction of the energy minimization and speed of the model nodes is the resultant of all component energy gradients:

12 $$\begin{aligned} \frac{\partial E}{\partial c\left[ k \right]} & = \mathop \int \limits_{\varGamma }^{{}} \left( {\overline{g} \left( x \right)\beta^{d} \left( {\frac{x}{h} - k} \right) + \overline{{E_{\text{S}} }} \left( x \right) + \overline{{E_{\text{B}} }} \left( x \right)} \right){\text{d}}x \\ & = \mathop \int \limits_{\varGamma }^{{}} \left( {\gamma \left( x \right)\overline{g} \left( x \right)\beta^{d} \left( {\frac{x}{h} - k} \right) + \alpha \left( x \right)\left| {\frac{{{\text{d}}\varGamma }}{{{\text{d}}x}}} \right|^{2} + \beta \left( x \right)\left| {\frac{{{\text{d}}^{2} \varGamma }}{{{\text{d}}x^{2} }}} \right|^{2} } \right){\text{d}}x, \\ \end{aligned}$$

where $\alpha \left( x \right)$, $\beta \left( x \right)$ and $\gamma \left( x \right)$ weigh the influence of the different components on the overall energy.

### Segmentation

The segmentation algorithm works similarly as the one presented in [22]. In each iteration, it calculates the overall model energy as well as direction and speed of modification of the model. Modification vectors for all model nodes are multiplied by a step factor $\lambda$ before being applied. If a step leads to energy decrease, $\lambda$ is multiplied by a speed increase factor $\lambda_{\text{d}}$ and the algorithm moves to the next iteration step. If the step leads to energy increase, the model modification is not applied and $\lambda$ is divided by a speed decrease factor $\lambda_{\text{z}}$. The algorithm is stopped either after a maximal number $\left( {\hbox{max} I} \right)$ of iterations has been reached, or if the energy could not be reduced by defined number $\left( {\hbox{max} I_{\text{z}} } \right) {\text{of trials}}$. To balance the role of the different energy components, each of them is normalized to the range [0,1] using the following formula:

13 $$E_{k}^{\prime } \left( x \right)\; = \;\frac{{E_{k} \left( x \right) - E_{k{\min} } }}{{E_{k{\max} } - E_{k{\min} } }},\;\;\; k \in \left\{ {I,S,E} \right\},$$

where $E_{k{\min} }$ and $E_{k{\max} }$ are minimum and maximum values of the energy across the whole model. Taking (13) into consideration, Eq. (11) gets the following form:

14 $$E^{\prime } \; = \;E_{\text{I}}^{\prime } \; + \;E_{\text{S}}^{\prime } \; + E_{\text{B}}^{\prime } .$$

The model and algorithm have been implemented in the Java programming language within Pmod PCARD tool [25]. As the left ventricular wall needs to be segmented, the method is applied to epicardium and endocardium segmentation separately. The space between these segments is considered as the myocardium segment. The implementation puts both epicardial and endocardial models in the same coordinates system, i.e. point $r = 0$ is common for both of them. It also supports mutual restrictions of the models, preventing one model from moving too far from another one. In practice, endocardium segmentation is performed first. In the subsequent epicardial segmentation, the r coordinates of the model are restricted to a physiologically justified wall range. This restriction is required because the epicardial wall is often not well delineated due to signal from neighboring organs (e.g. liver), or due to lack of signal in regions with abnormal perfusion (e.g. because of infarct scar).

Parameters describing contribution of different types of energy to the overall value (*α*, *β*, *γ*) were calculated using the routine based on the Powell’s method of function minimization [30]. To find the best fitting parameters, a series of segmentations with *α*, *β* and *γ* values changing within the range [0,1] were performed and their results were compared to the reference segments outlined manually. Quality of the segmentation was quantified using Dice index [31] between the found myocardium segment and a reference one. The minimized cost function was defined as the one’s complement of the Dice index, i.e. 1-Dice index. The starting point of the minimization algorithm was set to *α* = 0.5, *β* = 0.5 and *γ* = 1 with the default step for all three values 0.1. The calculated energy contribution parameter values as well as the other method parameters are listed in Table 1.

**Table 1:** Segmentation method parameters used for the validation

Parameters		Epicardium	Endocardium
max*I*	Maximum number of iterations	100	100
max*I*_z_	Maximum number of iterations with no energy decrease	5	5
*λ*	Algorithm step factor	1.3	1.3
*λ*_d_	Speed increase factor	1.1	1.1
*λ*_z_	Speed decrease factor	1.1	1.1
*d*	B-spline function degree	2	2
*N* × *M*	Number of model surface nodes (*Φ* × *θ*)	36 × 18	18 × 9
*α*	Model stiffness weight	0.4	0.4
*β*	Model bending resistance weight	0.6	0.6
*γ*	Image energy weight	1	1
*S*	Side of a cubic neighborhood in which the local image energy is calculated	20 [mm]	20 [mm]
*M*_L_	Approximate length of left ventricle long axis	75 [mm]	
*M*_R_	Approximate left ventricle base radius	30 [mm]	
*M*_W_	Approximate thickness of left ventricle muscle	10 [mm]	

Epicardium, parameters used for epicardial segmentation; endocardium, parameters used for endocardial segmentation

**Fig. 1:**
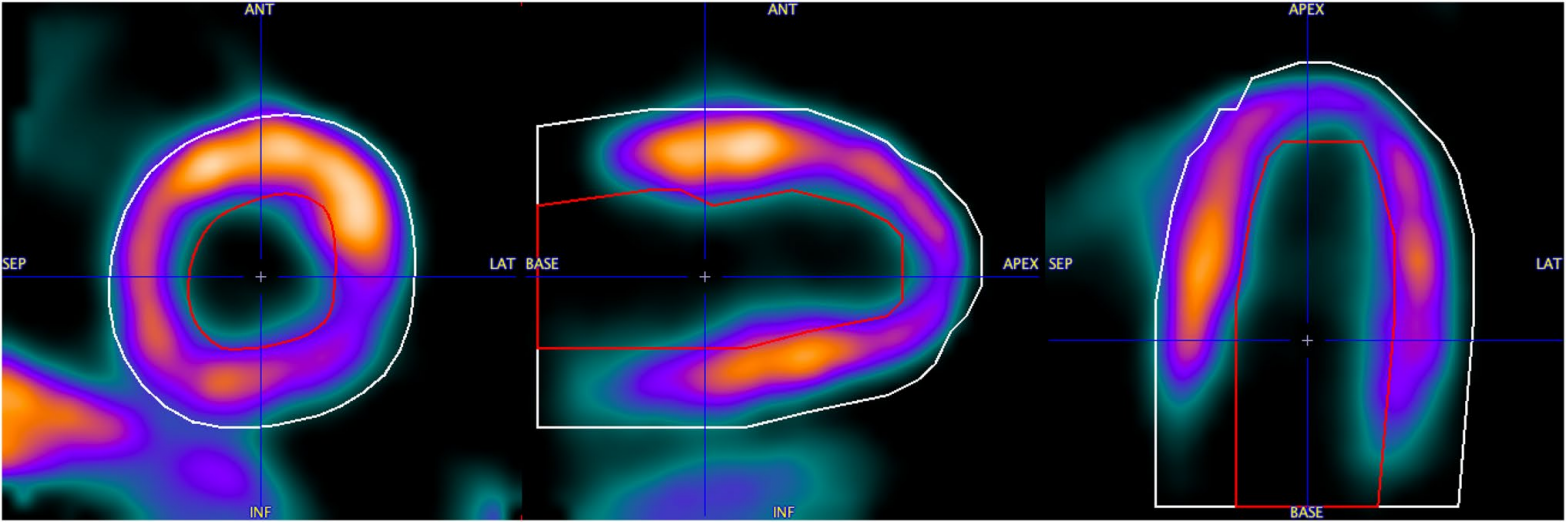
Pre-segmentation results on gated PET data averaged across all gates. Epicardial (white contour) and endocardial (red contour) segments shown in the figure are input for the proposed segmentation method

**Fig. 2:**
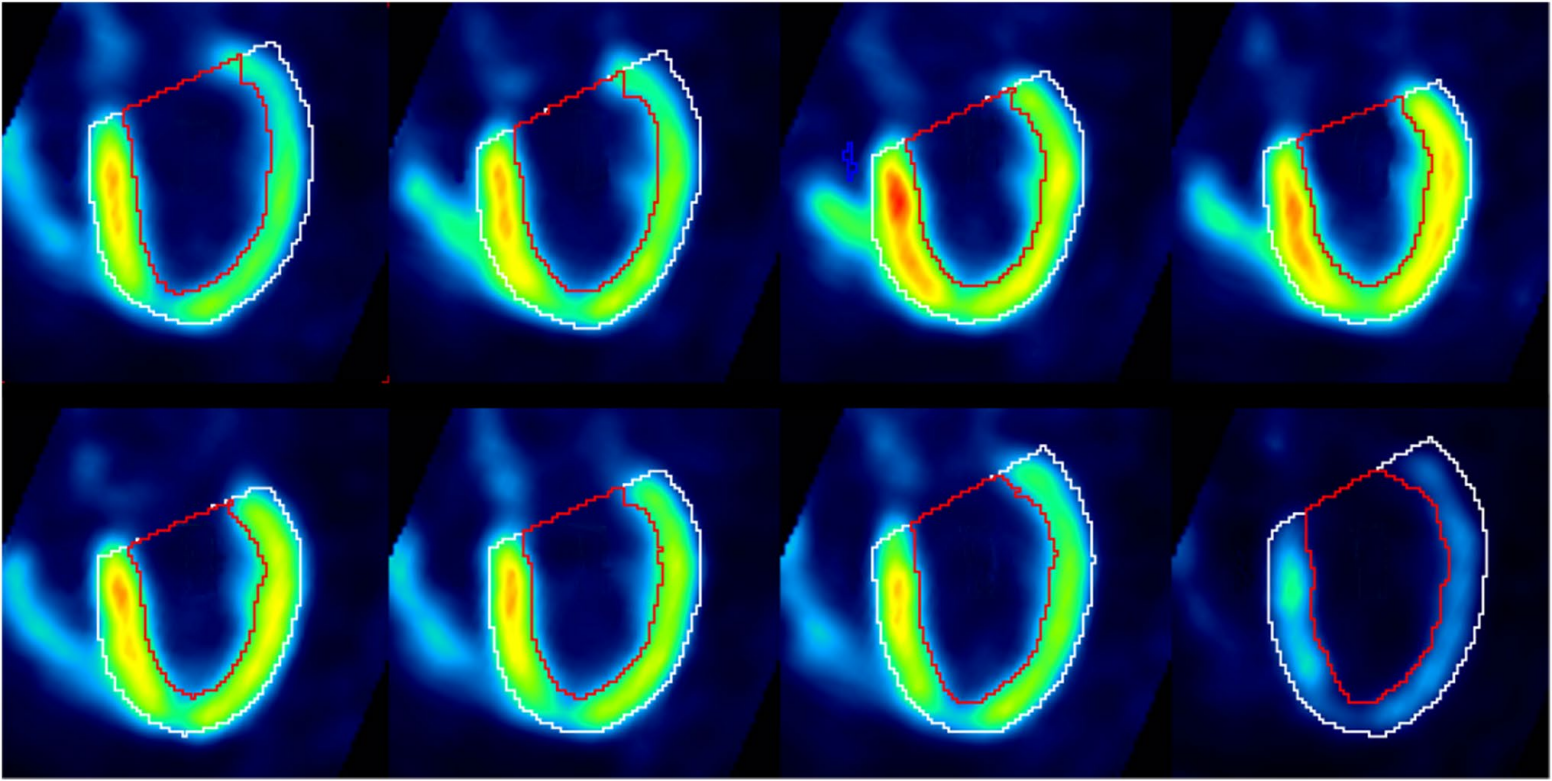
Segmentation results for cardiac gated PET data (eight equal bins)

The proposed segmentation method requires an initial model definition. In the PCARD tool a separate algorithm obtains it. It requires a priori setting of parameters describing the left ventricle approximately: long axis length, base radius and wall thickness. Furthermore, it requires that the data are resliced to short-axis orientation, with the heart long axis along the *z*-direction. The wall approximations are obtained by tracing rays from the long axis and analyzing the uptake profiles along them. An example of the resulting epi- and endocardial segments outlined on gated PET data averaged across all gates is shown in Fig. 1. They form the input for the energy-based segmentation described above. An example of the results applied to human gated PET data (eight bins) is shown in Fig. 2.

**Fig. 3:**
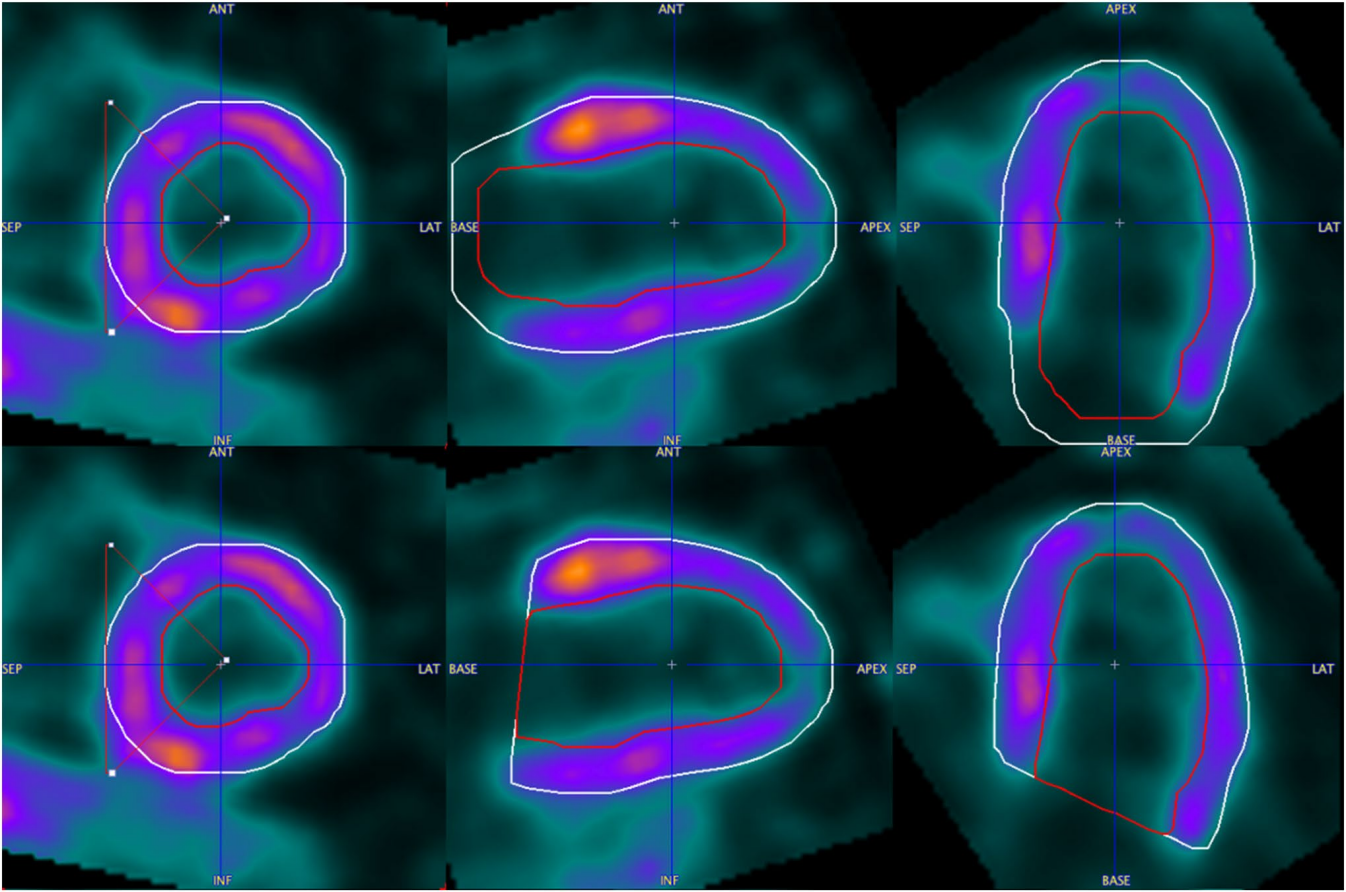
Epicardial and endocardial segments just after model segmentation (upper) and restriction (lower) to the valve plane

In PET data, the valve that closes the ventricular base is not visible. Therefore, additional post processing is required to approximate the valve plane. The following algorithm was implemented. First a mask of the myocardium is obtained using the Otsu threshold determined from the pixel histogram [32]. Having the data oriented in the short-axis projection, the center of mass in each slice between the apical and the basal plane is determined from the mask. From each center, rays are cast at equal angles. For each angle, the most basal slice is determined, for which the ray intersects with the mask. It is assumed that the intersection point in the middle of the mask wall belongs to the valve plane (valve point). The valve points in all angular directions provide an approximation of the valve plane. Hence, a plane is fitted to all valve points. An example of the initial segmentation after trimming by the fitted valve plane is shown in Fig. 3.

### Validation

To validate the new segmentation method, clinical as well as phantom data were processed and the results compared to those obtined using two software packages widely used in the clinical practice, QGS—Quantitative Gated SPECT, Cedars-Sinai Medical Center [26], and Emory Cardiac Toolbox (ECTb), Emory University Medical Center [27].

The new method was incorporated in the Pmod PCARDP Tool, version 3.902. It represents a localized version [19] calculating image energy $E_{\text{I}}$ (2) only in a cubic neighborhood of the current node.

The phantom data obtained during acquisition of two left ventricle phantoms of a known volume were analyzed. The phantoms were: an anthropomorphic phantom (Heart/Thorax Phantom, RSD Inc.) and a simplified isolated ventricular phantom Cardiac Insert Model ECT/CAR/I (BIODEX Medical Systems). The first phantom will be further referred to as the phantom A and the latter will be referred to as the phantom B.

The phantom A was filled with ^18^FDG, using a total activity of 37 MBq (1 mCi). To reproduce the proportions of activity usually observed in a human study, the heart wall (including the three defects provided by the producer to simulate perfusion defects) was filled with a concentration similar to the liver, whilst the concentration of the cardiac cavities (atria and ventricles) was one-fourth and that of the thorax one-tenth; finally the lungs were kept free of activity. After completion of the filling procedure, the phantom was laid on the PET/CT Gemini TOF scanner couch and the acquisition was performed. A CT study for attenuation correction was collected and then the data were acquired using the gated PET protocol, with the electrodes connected to a volunteer outside the scanner room to simulate the ECG signal for gating. The gated PET acquisition was reconstructed using the iterative method 3D-RAMLA (45 slices, matrix 144 × 144, voxel dimensions: 4 × 4 × 4 mm) with two iterations and three subsets, after decay, attenuation, random, scatter and time of flight correction. Before the final reconstruction of the images, the alignment of CT and PET images was controlled and corrected if necessary. Since the producer provides just the total volume of the cardiac cavities, the measurement of the left ventricular volume was performed by weighing the empty heart insert with a high precision balance, and then repeating the measurement after having slowly filled the sole left ventricular cavity with colored water through the proper injection hole, taking care of avoiding the overflow of the water in the nearby right ventricular cavity that communicates with the left one above the septum. The resulting volume was 75 ml.

The wall cavity of the phantom B was filled with 185 MBq (5 mCi) of ^18^FDG, and the images were acquired with a standard gated PET protocol for 10 min on a Discovery 710 PET scanner (GE) and then iteratively reconstructed with VUE point HD (18 subsets, two iterations). The ventricular cavity volume, reported by the producer to be about 60 ml, was measured as above described and found to be exactly 63 ml.

Besides the phantom data, a patient population of 60 patients, who had undergone cardiac PET scanning in both stress and rest conditions, has been analyzed. In particular, there were 29 patients studied because of suspected microvascular dysfunction in the setting of hypertrophic or dilated cardiomyopathy, or of secondary left ventricular hypertrophy, and 31 patients studied because of known or suspected coronary artery disease. Within the population, there were no patients with wide and severe resting or inducible perfusion defects.

Patients were studied with a standard resting and stress protocol using a PET/CT Gemini TOF scanner (Philips) as previously described [33]. Patients were submitted to CT imaging for attenuation correction, followed by resting study with administration of 370 MBq of ^13^NH_3_ in slow bolus and dynamic list mode acquisition lasting 9 min. Immediately thereafter, an eight-frame gated PET acquisition was started for additional 5 min. After 60 min, the stress study was performed using similar modalities, with the administration of 0.56 mg/kg of dipyridamole over 4 min. After 3 min of dipyridamole completion, 370 MBq of ^13^NH_3_ was injected and a second dynamic study acquired, again followed by a gated PET acquisition. No electrocardiographic or respiratory gating was applied. The study was then reconstructed using the same modalities described above for the phantom studies. Rest and stress end-systolic and end-diastolic volumes were obtained using the software mentioned above.

## Results

### Physical models

The phantom data were analyzed using QGS and PCARD using the proposed algorithm. The results compared to the real phantom volume are presented in Table 2. The mean relative error between QGS measurements and the physical volume is 6.66% and the mean relative error for PCARDP is 3.59%.

**Table 2:** Estimated volume measurements compared with the true phantom volumes (63[ml] and 75[ml])

Phantom	PCARD		QGS [ml]	
	Volume [ml]	Error [%]	Volume [ml]	Error [%]
A. 63 [ml]	65	3.17	63	0.00
B. 75 [ml]	78	4.00	85	13.33

*Volume* volume measurement, *Error* relative error compared to the physical volume

### Clinical data

**Fig. 4:**
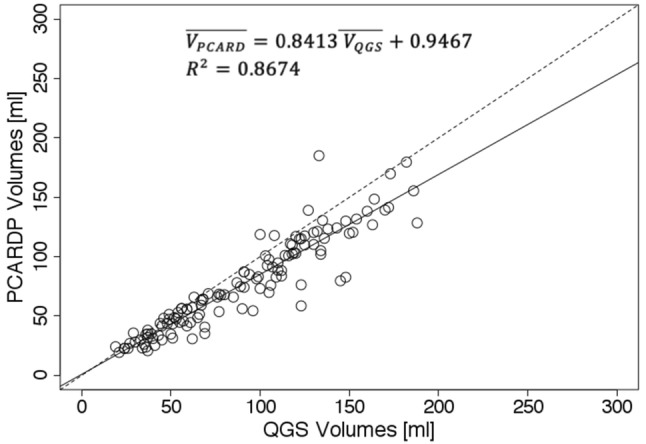
Scatter plot of left ventricle volumes obtained using Pmod PCARD and QGS software. Dashed line represents identity line. Plain line represents the linear regression line expressed by the following equation: $\overline{{V_{\text{PCARD}} }} \; = \;0.8413\overline{{{\text{V}}_{\text{QGS}} }} \; + \;0.9467$. $\overline{{V_{\text{PCARD}} }}$ is volume obtained using Pmod PCARD. $\overline{{V_{\text{QGS}} }}$ is volume obtained by QGS. R^2^ is the coefficient of determination

**Fig. 5:**
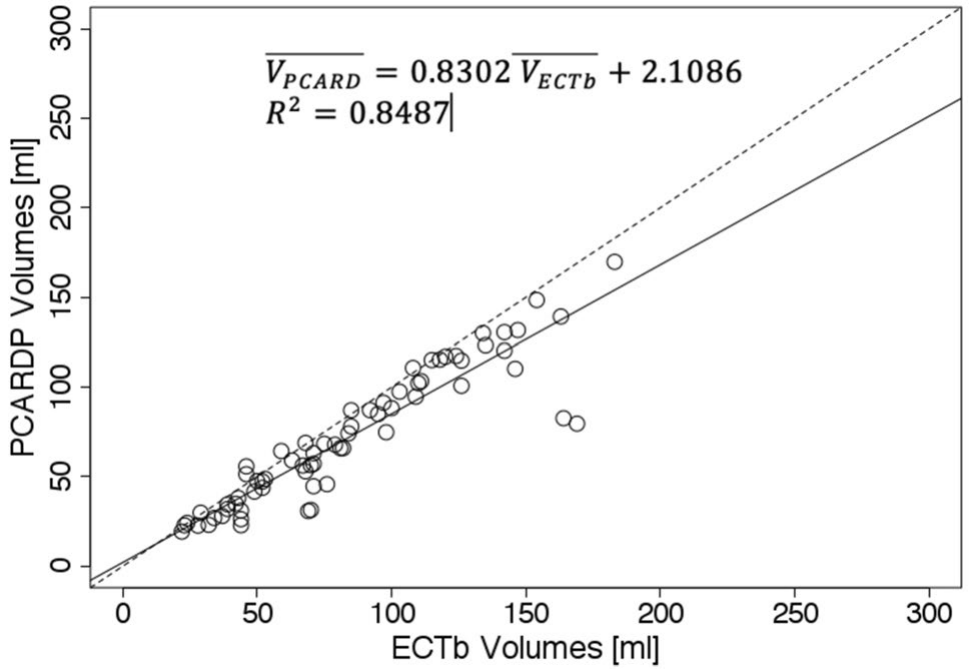
Scatter plot of left ventricle volumes obtained using Pmod PCARD and ECTb software. Dashed line represents identity line. Plain line represents the linear regression line expressed by the following equation: $\overline{{V_{\text{PCARD}} }} \; = \;0.8302\overline{{V_{\text{ECTb}} }} \; + \;2.1086$. $\overline{{V_{\text{PCARD}} }}$ is volume obtained using Pmod PCARD. $\overline{{V_{\text{ECTb}} }}$ is avolume obtained by ECTb. *R*^2^ is the coefficient of determination

**Fig. 6:**
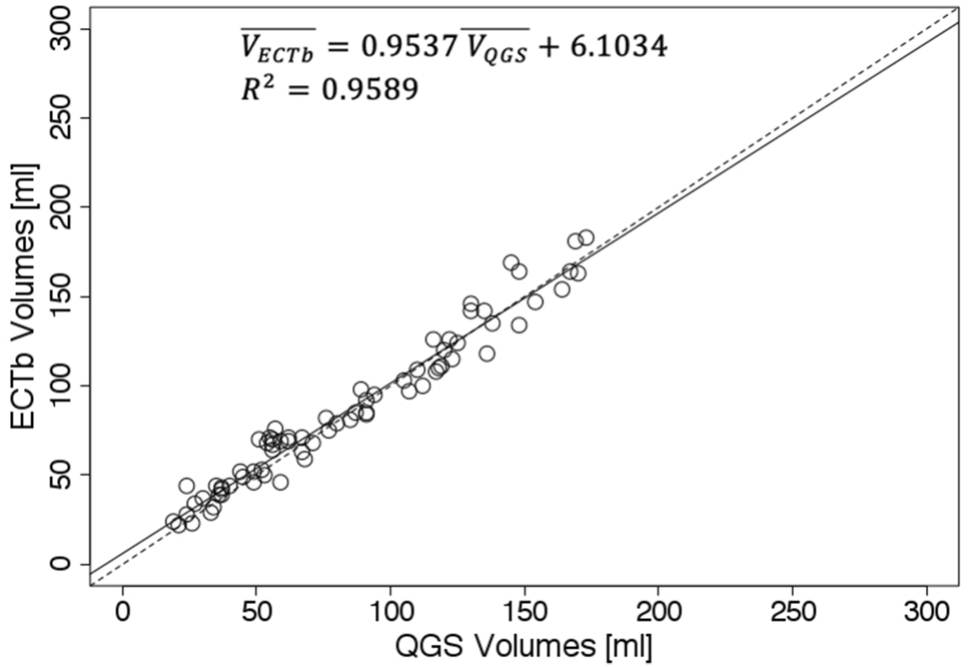
Scatter plot of left ventricle volumes obtained using Pmod ECTb and QGS software. Dashed line represents identity line. Plain line represents the linear regression line expressed by the following equation: $\overline{{V_{\text{ECTb}} }} \; = \;0.9537\overline{{V_{\text{QGS}} }} \; + \;6.1034$. $\overline{{V_{\text{ECTb}} }}$ is volume obtained using ECTb. $\overline{{V_{\text{QGS}} }}$ is volume obtained by QGS. *R*^2^ is the coefficient of determination

For logistic reasons, it was not possible to analyze all 60 patients using all three programs. The following pair-wise comparisons were feasible: PCARD compared to QGS (31 patients), PCARD compared to ECTb (18 patients), ECTb compared to QGS (18 patients). For each patient, two volumes (EDV, ESV) were calculated under stress and rest conditions, resulting in four observations for each patient. The results are shown as scatter plots in Figs. 4, 5 and 6. Each scatter plot presents the comparison of the measured LV volume between two programs as well as the result of the linear regression analysis performed using the least squares method. All pair-wise comparisons result in *R*^2^ higher than 0.8 and slope direction slightly smaller than 1.

**Fig. 7:**
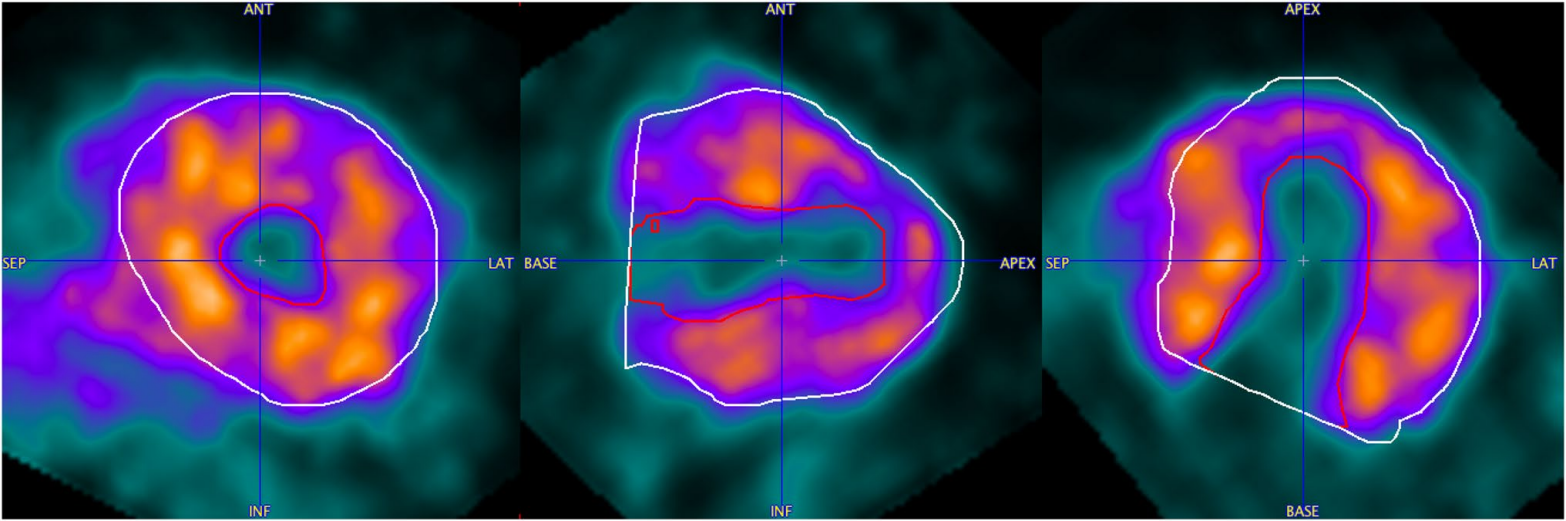
Segmentation result in the end-systolic frame for the case with severe form of hypertrophic cardiomyopathy (HCM) in the dilative phase. Epicardial (white contour) and endocardial (red contour) segments are shown

**Fig. 8:**
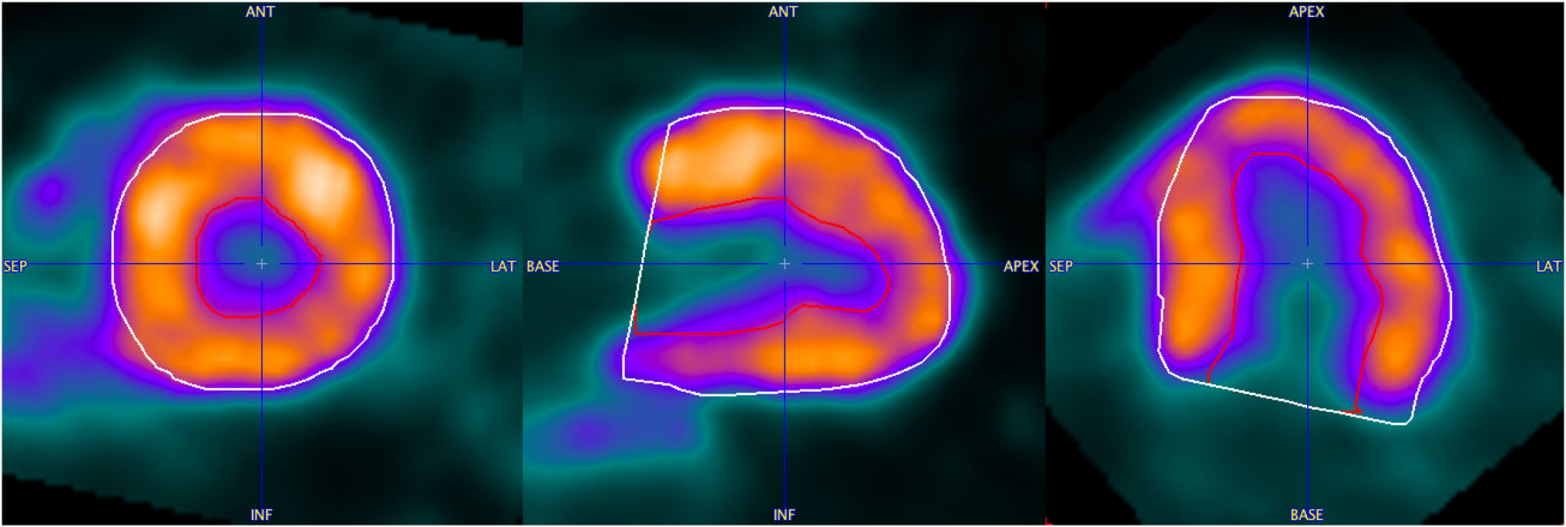
Segmentation result in the end-systolic frame for the case with septal hypertrophy. Epicardial (white contour) and endocardial (red contour) segments are shown

**Fig. 9:**
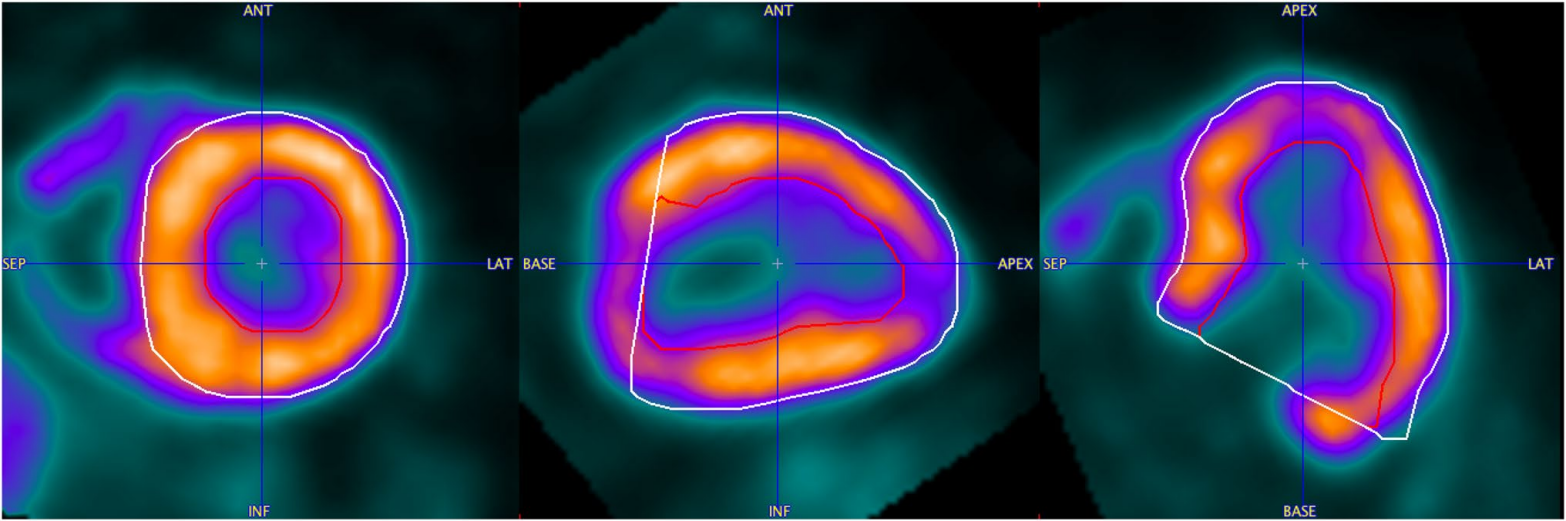
Segmentation result in the end-systolic frame for the case with septal hypertrophy. Epicardial (white contour) and endocardial (red contour) segments are shown. The segmentation inaccuracy can be observed in the lateral basal sectors

Some points on the scatter plots are visibly more distant from the regression line than the points. All of them are the examples of not healthy patients. In Fig. 7, the data of a patient with a severe form of hypertrophic cardiomyopathy (HCM), already in a dilative phase, are shown. In Figs. 8 and 9, examples of patient data with septal hypertrophy are displayed. Although the patient suffers from a septal hypertrophy, the segmentation results are correct in the septal sectors. In Fig. 9, the lateral basal sector inaccuracy shows underestimation of the left ventricular volume.

## Discussion

The presented scatter plots and linear regression results demonstrate a good correlation ($R^{2} > 0.8$) between results obtained by the new segmentation method and the reference results obtained by clinically used software. The linear regression equations suggest that there is a scaling factor of approximately 0.84 between PCARD and the reference results. It is unclear where this factor comes from. When processing phantom data, PCARD only slightly overestimated the true volumes, whereas QGS results were not consistent: for the simplified phantom QGS estimated the exact volume, whereas it overestimated the volume by 13% for the anthropomorphic phantom.

Correlation between QGS and ECTb is better than with PCARD. It is notable, however, that three different data sets had to be used for the comparisons, and manual interventions in PCARD were restricted to the short-axis reorientation. The scatter plots indicate some clear outliers. In practice, in the case of an erroneous segmentation result due to unusual heart geometry, the geometric parameters axis length and base radius of the method would be adjusted to improve the segmentation output. In Figs. 7, 8 and 9, the data correlating to the outlier point can be seen. The segmentation results in Figs. 7 and 8 are favorable for the proposed segmentation method.

Among the limitations of the proposed method, one should mention the deficiency in valve plane fitting algorithm if there is lack of perfusion in the heart basal layer either on lateral or septal side. Such regions with low signal level, resulting from e.g. infarction may result in detected valve plane shift towards heart mid layer and in LV volume underestimation.

Despite existence of the scaling factor for absolute volume calculations, the proposed method can be applied for left ventricular gated PET analysis. Phantom experiments show that the volume values obtained using PCARD are in agreement with the real values.

This study was not conceived for assessing the clinical implications of the proposed segmentation method nor to evaluate possible advantages over other established approaches. However, since each of so-far implemented methodologies can face difficulties in specific patients, the availability of another segmentation method could be useful. The phantom measurements seem favorable for PCARD; however, due to the small number of measurements, the results are not conclusive.

The segmentation method is derived from the BEAS algorithm, dedicated for ultrasound imaging. Its development proposed in this paper made it appropriate for gated PET data. Further stiffness and elasticity parameters modifications may allow adjusting the method to other imaging modalities, such as magnetic resonance or computed tomography.

## Conclusions

The analysis of gated PET clinical data by the proposed method results in volume measurements comparable to established methods. As the phantom experiments also demonstrate that the volume values correspond to the physical ones, it can be concluded, that the proposed segmentation method can be recognized as a good alternative for the reference methods.
